# Mind the gap: Physicians’ assessment of patients’ importance weights in localized prostate cancer

**DOI:** 10.1371/journal.pone.0200780

**Published:** 2018-07-26

**Authors:** Katya Tentori, Stefania Pighin, Claudio Divan, Vincenzo Crupi

**Affiliations:** 1 Center for Mind/Brain Sciences, University of Trento, Trento, Italy; 2 Urology Division of the Santa Chiara Hospital, Trento, Italy; 3 Department of Philosophy and Educational Sciences, University of Torino, Torino, Italy; Brown University, UNITED STATES

## Abstract

**Background:**

The management of localized prostate cancer is challenging because of the many therapeutic options available, none of which is generally acknowledged as superior to the others in every respect. The selection of the most appropriate treatment should therefore reflect patients’ preferences.

**Objective:**

The purpose of the following study was to pilot a new approach for investigating whether urologists who had previously provided patients with therapeutic advice actually knew their patients’ importance weights concerning the relevant aspects of the treatments at issue.

**Method:**

Participants were patients recently diagnosed with localized prostate cancer (*n* = 20), urologists (*n* = 10), and non-medical professionals (architects, *n* = 10). These last served as a control group for the urologists and were matched to them for age and gender. Patients’ importance weights were elicited by two standard methods (Direct Rating and Value Hierarchy). Each urologist was asked to estimate (with Direct Rating) his/her patient’s importance weights. The same task was performed by a corresponding architect, who never met the patient and knew only the patient’s age. Univariate and bivariate statistical analyses were performed to investigate the association between importance weights as elicited from patients and as estimated by urologists and architects, as well as to assess whether such agreement was attribute-dependent.

**Results:**

Participants found both elicitation methods easy to use. The correlation between patients’ actual importance weights and urologists’ estimates was poor and comparable to that obtained between patients and architects. This result did not depend on the attribute considered, with the sole exception of the attribute “Effectiveness in curing the cancer”, which was evaluated as the most important attribute by the majority of participants.

**Conclusion:**

These findings demonstrate the feasibility of the employed methodology and highlight the need to support preference-sensitive decisions in clinical practice by facilitating the elicitation of patients’ importance weights, as well as their communication to physicians.

## Introduction

Some medical decisions are clear-cut because there is an effective gold standard that does not have major drawbacks. In other situations, significant trade-offs have to be made among all relevant aspects of the available options (e.g., benefits vs. side effects of competing treatments). In these cases, personal considerations can make a difference, and, given the same health conditions, what is better for one individual is not necessarily so for others. Involving patients in such preference-sensitive decisions has been widely advocated, especially in the case of life-threatening illnesses and/or treatments with risky outcomes [1–5]. It is not just a matter of ethics: patient participation has been shown to be associated with better understanding of medical information, increased satisfaction with care, greater adherence to therapy, improved health outcomes, lower anxiety and discomfort, as well as reduced costs of care [5–8]. However, despite general agreement on the importance of engaging patients in active decision making about their health care, and despite efforts to educate them in how to play this role, implementation has proved challenging [9–15]. Furthermore, patients differ widely in the extent to which they feel comfortable with being in control of their own health decisions, depending on various factors, such as their mastery of medical terms and concepts, physical and cognitive ability, emotional state, beliefs, previous experiences with health services, and last but not least, their perception of the adequacy of information received [12,16–19]. In the end, although most patients today are less likely to delegate medical decisions, they do make their choices after seriously considering their physicians’ recommendations [20–23]. It therefore appears important to determine how well-informed these recommendations are when it comes to preference-sensitive decisions. The present study is aimed at proposing a new procedure for enhancing the understanding of this topic through a focus on treatment decisions for clinically localized prostate cancer.

Prostate cancer (hereinafter, PCa) is one of the most common cancers among adult males [24,25], and a leading cause of cancer-related death, both in the USA [26] and Europe [27]. Clinically localized PCa is a good case study for our purposes in two ways. The first is the large number of available treatment options (including active surveillance, radical or nerve-sparing prostatectomy, external beam radiation therapy, brachytherapy, cryosurgery, and hormonal therapies), none of which is acknowledged by experts as the optimal choice in every respect for all patients [28]. Indeed, mortality rates associated with the various treatments are not significantly different [29], and each can involve significant side effects on quality of life [24,30], thus making this choice an exemplary (“archetypal,” [31]) preference-sensitive decision. The second reason to focus on clinically localized PCa is that older males newly diagnosed with cancer (especially those with a lower level of education) are among the patients most reluctant to engage in active decision making concerning their health [6,18,20,32–34]. This finding is in line with the results of various studies showing a major role of physicians in treatment decisions concerning clinically localized PCa: patients are not always given the opportunity to discuss their inclinations during clinical appointments and are more likely to receive the treatment favored or best known by their physician [35–41].

Even assuming physicians were capable of prioritizing patients’ preferences over what they think is in the patients’ best interest (itself not an easy task, as pointed out in [42]), it is unclear whether physicians actually know what patients with clinically localized PCa actually prefer. Somewhat surprisingly, there is not much earlier empirical work on this matter. A few studies have focused on specific aspects of the PCa treatment decision process and found, for example, that physicians’ substitute preferences and recommendations were strongly driven by their own inclinations and/or medical factors (such as patients’ age and Gleason score), to the detriment of patients’ interests in the less-toxic treatment [43] or in remaining sexually active [39]. In what is, to our knowledge, the most systematic study on the subject, Elstein and collaborators [44,45] documented scant agreement between physicians and patients on specific components of a multi-attribute model of localized PCa. Newly diagnosed patients had to both evaluate the utilities of selected health states and rank the importance of a number of attributes on which these health states were defined. Physicians had to provide their views of which utilities and importance rankings would be in the patients’ best interest. For both health states and attributes, the patient-physician correlations were variable and, in general, lower than the critical value (.8) that could justify substituted judgment, according to Elstein et al. [44,45].

While related to the previous investigations that examined physicians’ awareness of localized PCa patients’ preferences, the approach proposed in the present study differs from them in some important respects. First, we were interested in analytically exploring the importance of an extensive set of attributes rather than only a few isolated aspects (e.g., preservation of sexual function). Second, our focus was on treatment options rather than health states. We concur with Elstein et al. [44,45] that the desirability of various health states may affect patients’ treatment decisions, yet the two kinds of assessment have different implications. Indeed, even a perfect patient-physician agreement on health states would be fully compatible with different treatment choices if important aspects of the available treatments (e.g., their duration) were missing from the health state evaluation. Symmetrically, a strong disagreement on health states would not necessarily translate into different treatment choices, since the available treatments may be indistinguishable with respect to these states. For these reasons, we selected a set of attributes that allow direct and comparative assessment of the most common treatments for clinically localized PCa (a detailed description of the attributes, as well as an explanation of their structure, is provided in the Materials section and in S1 Appendix). The third major difference with previous studies is that the agreement between patients and physicians was evaluated by comparing it, in addition to a threshold, with the agreement obtained between patients and a control group of non-medical professionals who had no knowledge of the patients and were unfamiliar with PCa treatments. We think that such a control group of participants provides a suitable benchmark to evaluate the goodness of physicians’ estimates, since one can reasonably assume that these non-expert participants, who were blind to patients, were clearly unqualified to advise patients on their treatment decisions. The fourth and most important difference concerns the type of judgment elicited from physicians. Previous studies [43–45] showed that patients’ and physicians’ evaluations may diverge. However, this does not automatically translate into a disregard of patients’ preferences, especially because the medical community acknowledges that clinically localized PCa treatment decisions ought to reflect patients’ personal values. Accordingly, we did not measure the agreement between patients’ and physicians’ weights, but rather the agreement between patients’ weights and physicians’ estimates of those same weights. This allowed us to quantify the physicians’ awareness of their patients’ preferences independently of their own opinions, whether in application to themselves or in consideration of their patients’ best interest.

## Methods

### Participants

Participants in this study comprised 20 patients, 10 physicians, and 10 non-medical professionals. The patient group consisted of 20 men, mean age 67 years (*SD* = 7), newly diagnosed (i.e., within the preceding 4 weeks) with clinically localized PCa, staging T_1-2_N_0_M_0_. Patients had not yet started any treatment, had no significant comorbidities, and had a life expectancy greater than 10 years. Physicians were 10 urologists (2 female and 8 male), mean age 50 years (*SD* = 9), working at the urology units of two main clinical facilities in northeast Italy. Finally, the non-medical professional group consisted of 10 architects (2 female and 8 male), mean age 45 years (*SD* = 8).

#### Ethics statement

The study was approved by the research ethics committee of the APSS (the regional healthcare provider within the Provincia Autonoma di Trento, Italy). Written informed consent was obtained from all individual participants included in the study. All procedures were in accordance with the Helsinki declaration.

### Material

In accordance with the guidelines of Multiple Criteria Decision Analysis for Health Care [46], we generated our list of attributes so as to satisfy the following desiderata: to capture the complexity of the available clinically localized PCa treatments (*completeness*), to include only relevant and no double-counted attributes for the purposes of comparing existing PCa treatments (*non-redundancy* and *non-overlapping*), and to ensure that the values of one attribute would not affect, nor would they depend on, the values of other attributes (*mutual preferential independence*, see also [47,48]). This last is a fundamental condition of multi-attribute utility theory that prevents the interaction between attributes and allows the additive decomposition of the utility function. Its fulfillment was made easier by our focus on treatments rather than health states, since the values of treatments (e.g., their expected effectiveness or side effects) are typically fixed and independent from each other, while the same does not hold for most attributes expressing how the patient feels (e.g., mood or pain, as used in [44,45]). Overall, our analysis generated eight attributes pertaining to four macro-dimensions, as described in Table 1.

**Table 1:** Attributes of the treatments.

Macro-dimensions	Attributes
Effectiveness	Effectiveness in curing the cancer
	Effectiveness in curing cancer-related disorders (e.g. as urge in urination, muscle tension, …)
Tolerability	Duration
	Discomfort (e.g., hospitalization, pain, …)
Temporary side effects	Temporary urinary problems
	Temporary digestive problems
Permanent side effects and/or complications	Permanent erectile dysfunction
	Other permanent problems (e.g., anesthesia-related complications, …)

Each attribute was accompanied by a short description that included a specification of its worst and best levels (see S1 Appendix). This was motivated by the consideration that importance weights depend on the ranges of attributes [49]. Such clarification also helped us make the attributes more understandable to patients and architects, who might otherwise be unfamiliar with this decision problem. It also indirectly confirmed that our list of attributes made sense to physicians, given that none of them raised any concerns. Attributes were presented to participants in two different orders (a randomly chosen one and its reverse). The attributes were presented to each patient-urologist/patient-architect pair in the same order.

### Procedure

Patients were assigned to urologists by the hospital administration based on the timing of the outcome of the biopsy and on the urologists’ shift schedule. Data were collected only after at least one meeting between urologist and patient, during which the urologist had informed the patient about the positive biopsy result, discussed with him the treatment options, and provided him with his/her own treatment advice. Since physicians were interviewed only after they had already advised a patient with a specific treatment, it seems reasonable to assume that they should have been aware of the patient’s importance weights. To minimize possible memory interference, each half of the urologist-patient pair was interviewed within three days of the other, without any other encounter taking place between the two in the meantime. Even within such a small time window, whenever a urologist raised a concern about her/his memory of a specific patient whose importance weights s/he had been asked to estimate, we did not force her/him and simply did not include that pair in our data collection. This reduced the number of participants, but it also helped us to avoid interference with normal clinical activity. In particular, it attenuated the risk that urologists would alter their patterns of practice with patients due to their anticipation of our questionnaire. Each architect was matched for age and gender to a unique physician and was paired with the same patient(s). Architects never met the patients; they were told only a patient’s age and that he had received a recent diagnosis of clinically localized PCa.

After being introduced to the eight attributes, participants were asked to provide their judgments, that is, importance weights for patients and estimates of patients’ importance weights for urologists and architects. More specifically, participants in all three groups (patients, urologists, and architects) were asked to rate each attribute independently on a scale ranging from 0 (= “not important at all”) to 100 (= “extremely important”). This elicitation method is known as Direct Rating (hereinafter DR, [50]) and is a simple technique with relatively low demands on time. For patients only, we also employed a version of the Hierarchical Point Allocation technique known as Value Hierarchy (hereinafter VH, [51]), in which the attributes are grouped into fewer macro-dimensions (in our case, the eight attributes were arranged in pairs into four macro-dimensions, see Table 1); participants have to distribute 100 importance points among the macro-dimensions (in our case, four, as in Table 1) and then, they have to further distribute 100 importance points between the attributes within each macro-dimension (two in our case). Importance weights are calculated by aggregating the two judgments and rescaling the result (i.e., by multiplying the within- and between-dimension point scores obtained for each attribute, and dividing the result by 100). Given that hierarchical techniques involve a number of pairwise comparisons, they are supposed to reflect people’s propensity for relative judgments, as well as to help them organize complex goal structures into hierarchical clusters [52]. Patients’ weights were elicited first with DR and then with VH. We decided to use only DR with urologists because they were already familiar with the structure of the attributes under consideration and, furthermore, because it was not realistic for them to complete a lengthy interview during their working hours. On the other hand, the use of two different techniques with patients allowed us to assess the consistency of results. Indeed, although both methods yield a cardinal scale of importance, DR reportedly yields a lower spread of weights than VH [49].

### Statistical analysis

We performed descriptive statistics first, in order to summarize patients’ importance weights for each attribute. The general agreement between patients’ importance weights and their estimates as provided by urologists and architects was assessed by computing Kendall rank-order correlations. To determine whether these correlations exceeded the critical value of .8, one-sample *t*-tests were performed. Paired *t*-tests were used to determine if the agreement with patients’ importance weights differed significantly between urologists and architects. To quantify the evidence in support of the null hypothesis in these two comparisons, we computed the corresponding Bayes factors (using JASP 0.7.5.6; www.jasp-stats.org). We also converted participants’ judgements into ranks and, for each attribute, determined by means of a binomial test whether urologists’ and architects’ ranks in agreement with patients’ ranks significantly exceeded those in disagreement. Finally, we assessed by Kendall rank-order correlations the within-subject agreement in patients’ weights elicited using the two DR and VH methods.

## Results

Patients’ mean importance weights for each attribute are reported in Table 2. Unsurprisingly, “Effectiveness in curing the cancer” was the most important attribute (whatever the method used to elicit weights), while the lowest weights were given to “Discomfort” and “Temporary digestive problems” (with DR and VH method, respectively).

**Table 2:** Patients’ mean importance weights (and standard deviation) for each attribute.

Attribute	DR	VH
	*M* (*SD*)	*M* (*SD*)
Effectiveness in curing the cancer	21 (6)	40 (11)
Effectiveness in curing cancer-related disorders	12 (7)	9 (6)
Duration	12 (6)	8 (5)
Discomfort	9 (5)	8 (5)
Temporary urinary problems	14 (5)	7 (4)
Temporary digestive problems	10 (5)	5 (3)
Permanent erectile dysfunction	11 (5)	12 (7)
Other permanent problems	13 (7)	13 (7)

Weights are on a scale from 0 (= “not important at all”) to 100 (= “extremely important”). In order to make the weights obtained with the two methods comparable, those elicited with DR have been normalized to sum to 100.

As shown in Table 3, the overall agreement between patients and urologists across the eight attributes was poor, regardless of the method used to elicit importance weights with patients (Kendall’s tau, *M* = .33, *Md* = .27, and *M* = .34, *Md* = .37, for DR and VH method, respectively). Similar figures were obtained for the agreement between patients and architects (Kendall’s tau, *M* = .29, *Md* = .32, and *M* = .25, *Md* = .30, for patients’ weights elicited using the DR and VH method, respectively). (Note that, although we had 20 patient-urologist/patient-architect pairs, the above statistics employ only 10 values each because, whenever a urologist and the corresponding architect estimated the importance weights of multiple patients, the mean correlation coefficient was considered.)

**Table 3:** Mean, median, and range of the Kendall’s τ coefficients between patients and urologists and between patients and architects.

Statistics	Patients (DR)		Patients (VH)	
	Urologists (DR)	Architects (DR)	Urologists (DR)	Architects (DR)
*M*^a^	.33	.29	.34	.25
*Mdn*^a^	.27	.32	.37	.30
*Range*^a^	.06/.61	-.04/.60	-.17/.69	-.34/.64
*t*-test	*t*(9) = .49, *p* = .632		*t*(9) = .73, *p* = .483	
BF_01_	2.92		2.59	

^a^ Mean, median, and range values were taken before Fisher Z transformation of the correlation coefficients.

In order to run inferential statistics, we applied a Fisher Z transformation to normalize the distribution of correlation coefficients. The agreement between patients and urologists was revealed to be significantly lower than .8, the “high-enough” cut-off in correlation proposed by Elstein et al. [45], *t*(9) = -5.99, *p* < .001, *t*(9) = -3.87, *p* = .004, for patients’ weights elicited using the DR and VH methods, respectively. A second analysis of the accuracy of urologists’ estimates of patients’ importance weights was made by comparing the agreements in patient-urologist and in patient-architect pairs. These did not significantly differ, according to two paired *t*-tests [*t*(9) = .49, *p* = .632 and *t*(9) = .73, *p* = .483, for patients’ weights elicited using the DR and VH methods, respectively]. Bayes factor analysis indicated that the obtained data were 2.92 and 2.59 times more likely to occur under the null hypothesis compared to the alternative hypothesis (when patients’ importance weights were elicited by the DR and the VH methods, respectively).

To quantify the agreement on each attribute, we converted all participants’ judgments into ranks and then compared these ranks within all patient-urologist and patient-architect pairs. In particular, for each attribute, we considered two ranks to be in agreement if and only if they differed by at most one position. For example, suppose that the attribute “Effectiveness in curing cancer-related disorders” was second in a patient’s ranking. If the same attribute had a rank between 1 and 3 in his urologist’s evaluation, the two ranks were considered to be in agreement—otherwise they were not. Finally, we computed the fraction of ranks in agreement (see Table 4). (Again, whenever a urologist and corresponding architect estimated the importance weights of multiple patients, the mean fraction of agreement was considered.)

**Table 4:** Fraction of ranks in agreement between patients and urologists and between patients and architects for each attribute.

Attribute	Patient DR		Patient VH	
	Urologist DR	Architect DR	Urologist DR	Architect DR
Effectiveness in curing the cancer	.74	.88*	.86*	.90*
Effectiveness in curing cancer-related disorders	.27	.62	.35	.52
Duration	.22	.26	.48	.18
Discomfort	.47	.37	.29	.25
Temporary urinary problems	.22	.24	.18	.27
Temporary digestive problems	.39	.36	.46	.44
Permanent erectile dysfunction	.22	.41	.12	.45
Other permanent problems	.45	.32	.52	.21

* Significantly greater than 0.5 according to a binomial test (*p* < .05).

The agreement in rankings between patients and both urologist and architects was generally poor, regardless of the attribute. The sole exception is represented by the attribute “Effectiveness in curing the cancer”, for which the ranks in agreement significantly exceeded those in disagreement for all but the patient-urologist pairs in which patients’ weights were elicited using the DR method (*p*s = .021 and *p* = .109, binomial test, respectively).

Finally, the within-subject agreement in patients’ weights elicited using the DR and VH methods was moderate (Kendall’s tau, *M* = .55, *SD* = .29). This might be because, as reported in the literature, the distribution of weights elicited by VH had greater spread than those from DR (coefficients of variations = .98 and .51, for weights elicited using the VH and DR methods, respectively, *F*(318) = 18.3, *p* < .001).

## Discussion

Traditionally, theoretical and empirical research on physician-patient communication has focused on how to improve the flow of information from the former to the latter [7,53–57]. Patients’ understanding of medical terms and concepts, including the pros and cons of available treatments, as well as relevant statistical information, is no doubt needed to help them be fully informed and actively involved in their own care. However, when it comes to preference-sensitive decisions, this is not enough. Preference-sensitive decisions involve at least two experts: the physician, who masters the clinical evidence, and the patient, who knows better what matters most to him. Unless one makes the unrealistic assumption that, once informed, patients decide among the available treatments with complete autonomy, their relevant preferences should be shared with physicians.

In this study, we piloted and demonstrated the feasibility of a new methodological approach to determine whether patients’ importance weights are effectively conveyed from patients to physicians. Our results, although referring to a limited number of participants, suggest that urologists’ accuracy in estimating these weights was poor and not significantly greater than that of non-medical professionals who had never met the patients and knew only their ages, regardless of the elicitation method used. This was in spite of the fact that urologists felt confident enough to provide patients a precise therapeutic recommendation. With respect to earlier results, this finding provides more direct evidence of a dearth of communication between patients and physicians, because it clarifies that their possible disagreements in treatment choice may reflect not only a difference in opinion but also physicians’ lack of awareness of what patients prefer.

The reasons for the information gap between PCa patients and urologists about relevant importance weights fall outside the scope of this study and represent an interesting matter for future research. Our preliminary results along with those mentioned in the Introduction suggest that the problem may stem from the structure of patient-physician encounters (which are typically physician-driven) and from the difficulty of inferring others’ importance weights. It has been claimed that physicians need help in determining patient preferences [43], but how this help should be given is far from obvious. Indeed, according to two recent reviews [57,58], most existing decision aids for localized PCa fail to demonstrate substantial benefits, because they tend to provide patients with detailed information about the features of available treatments rather than addressing personal importance. A more advanced generation of decision tools, known as Explicit Values Clarification Methods [59–61], has been developed in order to help patients determine what matters most to them. However, there are no established best practices, and most of the techniques that have been used do not have a clear theoretical or empirical grounding [60]. Multiple Criteria Decision Analysis may guide new strategies for improving physicians’ accessibility to patients’ preferences by suggesting concrete means with which to elicit and share decision weights. For example, one possibility might be to implement a routine practice in which the treatment options are briefly introduced to patients along with an extensive description of their relevant attributes. Once the content and ranges of these attributes have been fully explained, patients could fill in a grid with their importance weights. Physicians could then describe more precisely how well the available treatments perform on each attribute and start collecting patients’ opinions on the attractiveness (or unattractiveness) of various treatments. Finally, patients’ overall assessments could be contrasted with the results of the weight elicitation procedure. When the evaluations are consistent, it would give all parties reassurance that patients have understood the medical information received and have effectively voiced their preferences. Otherwise, it would indicate that clarification and/or a more in-depth evaluation is required. Either way, it would assist physicians in understanding patients’ priorities and would provide them with a sound basis for possible recommendations. We expect that not only the treatment choice but also the overall medical relationship could benefit from a systematized elicitation of importance weights, since it could foster patients’ feelings that the treatment is personalized, with a consequent increase in compliance and satisfaction [62,63]. Note also that a similar procedure could be generalized beyond the PCa treatment dilemma to other kinds of preference-sensitive medical decisions.

Future large-scale empirical studies aimed at quantifying physicians’ awareness of patients’ importance weights or facilitating the elicitation and sharing of patients’ preferences might also consider some other methodological open issues. First, to date there has been little empirical work on the effects and robustness of various preference-elicitation procedures, especially in the medical field. The modest correlation between the DR and VH methods found in this study suggests it might be worth exploring this topic more thoroughly by analyzing which elicitation procedure could best capture patients’ importance weights in terms of coherence and test-retest reliability—all without imposing an excessive cognitive burden. Another relevant and still unexplored issue concerns the consistency of weight measurements over a long time span. We elicited patients’ importance weights between diagnosis and treatment choice. This enabled us to prevent the distortion in preference that typically follows important decisions (like those generated by consolidation processes [64]), as well as to avoid potential confounds that might be introduced by the consequences of ongoing treatments, in particular the outcomes of care. However, some of the attributes that we considered (e.g., those referring to permanent conditions, like erectile dysfunction) also concern the distant future, and their evaluation may change in the course of the illness or upon its cure [65]. Accordingly, it might well be that the corresponding weights could be over- or under-estimated when choosing the treatment. Indeed, a number of studies have shown that people tend to make various errors when predicting their future preferences and feelings (a phenomenon known as faulty affective forecasting; for more on this, see [66–68]), with potentially strong implications for medical decision making. In principle, the observed gap could be accounted for, at least partially, by assuming that experienced physicians may have a better idea of what their patients would prefer in the long run than the patients have themselves. Longitudinal research is required to investigate this possibility and how importance weights may change over time, so that patients’ successive relevant preferences can be included in their treatment choices. Finally, future research could more thoroughly address the relationship between importance weights and choice. Knowing the relevant importance weights is a necessary but not sufficient condition for identifying the best treatment. Other factors may affect the preferences. For example, a patient may well decide to disregard all treatments whose values fall below a threshold on some crucial attribute, while being happy to compare the remaining options by summing their weighted values across the attributes. To the best of our knowledge, this compelling research topic has not been fully explored in the medical literature on preference-sensitive decisions.

## Conclusion

The results of this pilot study suggest that, although urologists typically have an active role in PCa treatment decisions, they may not be aware of patients’ importance weights concerning the relevant dimensions of the available options. While awaiting the results of more definitive studies, we might consider why this happens and how we could change routine procedures to facilitate the elicitation and sharing of this kind of information. At present, the laws of many Western countries grant patients the right to consent to or refuse the treatments that physicians recommend, yet they do not require the physicians to elicit patients’ preferences or to support them in a process of deliberation (see [5]). Interestingly, this does not hold for investment advisors, who are required to make a reliable assessment of their clients’ risk profile and financial objectives before providing them with suggestions (see the suitability requirements of the Markets in Financial Instruments MiFID II—Directive 2014/65/EU [69]). Unless we consider uninformed recommendations concerning health less problematic, similar measures in healthcare settings seem worthy of consideration.

## Supporting information

S1 Appendix Description of the eight attributes used in the study. (All materials are translated from Italian).
(PDF)
