# Supplementary material for: Mind the gap: Physicians’ assessment of patients’ importance weights in localized prostate cancer
Source: PLoS One. 2018 Jul 26;13(7):e0200780. doi: 10.1371/journal.pone.0200780 (PMC6062014; doi:10.1371/journal.pone.0200780)
Supplement: S1 Appendix — (All materials are translated from Italian). (PDF) [file pone.0200780.s001.pdf]

S1 Appendix

Description of the eight attributes used in the study. For each attribute, the minimum and the maximum levels were specified. (All materials are translated from Italian.)

---

1. **EFFECTIVENESS IN CURING THE CANCER**

   The effectiveness of alternative treatments in curing the cancer ranges between the following extremes:

   - In the best possible case, the cancer is conclusively defeated and the patient's life expectancy corresponds to that of a person in the same health condition but without the cancer.
   - In the worst possible case, the cancer is not defeated and the patient's life expectancy corresponds to that of a person in the same health condition who does not undergo any treatment.

2. **EFFECTIVENESS IN CURING CANCER-RELATED DISORDERS**

   The effectiveness of alternative treatments in curing cancer-related disorders ranges between the following extremes:

   - In the best possible case, all possible disorders associated with the cancer (e.g., post-void residual; urgency, frequency and difficulty in urination; infections) definitively disappear in the medium-long term, and the urinary system returns to a normal level of functionality.
   - In the worst possible case, all possible disorders of the urinary system associated with the cancer are not countered and they follow their normal course, as would happen in the absence of any treatment.

3. **DURATION**

   The duration of alternative treatments ranges between the following extremes:

   - In the best possible case, the treatment requires a few days.
   - In the worst possible case, the treatment requires going to the hospital daily over some weeks.

4. **DISCOMFORT**

   The discomfort of alternative treatments ranges between the following extremes:

   - In the best possible case, the treatment does not require hospitalization or catheterization, and it is painless.
   - In the worst possible case, the treatment requires up to three weeks of hospitalization and catheterization, with pain occurring in the affected areas.

5. **TEMPORARY URINARY PROBLEMS**

   Temporary urinary problems due to alternative treatments range between the following extremes:

   - In the best possible case, no temporary urinary problem occurs.
   - In the worst possible case, temporary urinary problems occur (e.g., incontinence, increase in the urinary frequency), for several months after the treatment.

6. **TEMPORARY DIGESTIVE PROBLEMS**

   Temporary digestive problems due to alternative treatments range between the following extremes:

   - In the best possible case, no temporary digestive problem occurs.
   - In the worst possible case, temporary digestive problems occur (e.g., diarrhea), for several months after the treatment.

7. **PERMANENT ERECTILE DYSFUNCTION**

   The impairment of sexual function due to alternative treatments ranges between the following extremes:

   - In the best possible case, sexual function is not affected at all by the treatment.
   - In the worst possible case, sexual function is definitively compromised in terms of a permanent erectile dysfunction.

8. **OTHER PERMANENT PROBLEMS**

   Permanent complications due to alternative treatments ranges between the following extremes:

   - In the best possible case, the treatment does not have any permanent negative side effect.
   - In the worst possible case, severe problems (e.g., anesthesia-related complications) arise during the treatment with permanent negative side effects (e.g., reduction in mobility and, in rare cases, death).
